# Highly Sensitive H_2_S Gas Sensor Based on a Lead-Free CsCu_2_I_3_ Perovskite Film at Room Temperature

**DOI:** 10.1021/acsomega.3c07694

**Published:** 2023-12-11

**Authors:** Kai Ou, Yue Wang, Wenting Zhang, Yongliang Tang, Yuxiang Ni, Yudong Xia, Hongyan Wang

**Affiliations:** School of Physical Science and Technology, Southwest Jiaotong University, Chengdu, Sichuan 610031, China

## Abstract

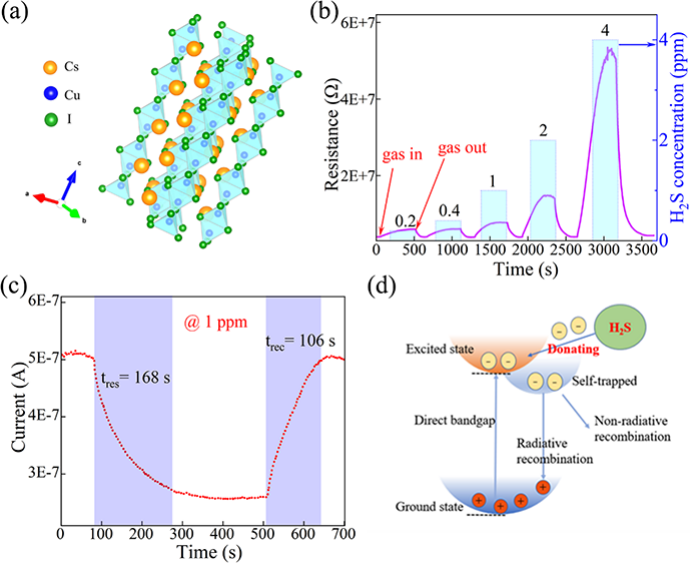

Recently, there have been reports of lead halide perovskite-based sensors demonstrating their potential for gas sensing applications. However, the toxicity of lead and the instability of lead-based perovskites have limited their applications. This study addressed this issue by developing a H_2_S gas sensor based on a lead-free CsCu_2_I_3_ film prepared using a one-step CVD method. The sensor demonstrated excellent sensing properties, including a high response and selectivity toward H_2_S, even at low concentrations (0.2 ppm) at room temperature. Furthermore, a reasonable sensing mechanism was proposed. It is suggested that the sensing mechanism sheds light on the role of defects in perovskite materials, the impact of H_2_S as an electron donor, and the occurrence of reversible chemical reactions. These findings suggest that lead-free CsCu_2_I_3_ has great potential in the field of H_2_S gas sensing.

## 1 Introduction

Gas sensors play an undeniable role in various fields, including medical, indoor, and outdoor environments, the automotive industry, and military monitoring systems. With the increase in industrial activities, the presence of toxic gases and volatile organic compounds has led to a decline in the air quality,^1,2^ such as NH_3_, H_2_S, CO_2_, CH_4_, NO_*x*_, and CO. Among these, H_2_S is an odorous, colorless, toxic and flammable gas, which shows a major threat to human health.^3^ The threshold limit value for the H_2_S concentration in ambient air is only 10 ppm. H_2_S gas can cause eye irritation at concentrations of 20 ppm and can lead to cardiovascular system breakdown and lung and nervous system damage at concentrations above 300 ppm. Furthermore, studies have linked even parts per billion (ppb) levels of H_2_S to health problems such as cardiovascular disease.^4^ Therefore, accurate monitoring of environmental pollutants, especially low concentrations of H_2_S, is essential to protect humans and animals from toxic gases.

The use of gas sensors in field applications is limited by factors, such as high detection limits, operating temperatures, and costs. Various approaches for monitoring H_2_S gas have been reported, including metal oxide semiconductors (MOSs), two-dimensional nanostructured materials, polymer, calorimetric, and so on.^5−8^ These approaches mainly focus on different nanostructures and materials. Typical MOS sensors and their heterojunction, such as SnO_2_,^9^ ZnO,^10^ TiO_2_,^11^ a-Fe_2_O_3_,^12^ CuO,^13^ and WO_3_,^14^ have been extensively studied for H_2_S gas detection due to their chemical stability, electrochemical activity, and excellent electron communication capabilities. However, most metal oxide sensors require high operating temperatures (200–500 °C). In recent years, based on structural and material innovations, some metal oxide H_2_S sensors have achieved high detection performance reaching parts per billion levels at room temperature.^15,16^ In addition to further optimizing metal oxides, new materials with high sensitivity and strong selectivity at room temperature have been researched. For example, graphene oxide,^17^ 2D transition-metal carbides and nitrides are appealing candidates for gas sensing due to good conductivity and abundant surface functional groups. Komsa et al. reported on a negative response of pristine Ti_3_C_2_T_*x*_ thin films for H_2_S gas sensing and proposed a reliable sensing mechanism based on experimental and theoretical calculations.^18^

Metal halide perovskite has emerged as a promising optoelectronic material due to its adjustable band gap, high optical absorption coefficient, low exciton binding energy, and high carrier mobility.^19,20^ The most representative metal halide perovskite has made many breakthroughs in the field of solar cells^21^, light-emitting devices,^22^ photodetectors,^23^ and electrochemical biosensors.^24^ In addition, metal halide perovskite sensors also exhibit high response, selectivity, fast response/recovery time at room temperature, and acceptable stability at low concentrations, attracting considerable attention from researchers.^25^ Many perovskite-based devices have been demonstrated with extraordinary sensing performance to various chemical and biological species in both solid and solution states.^26^ For example, Maity et al. has achieved a lead-based organometal halide perovskite ammonia sensor with an ∼55% response for 1 ppm regime of NH_3_ gas under nitrogen conditions or air.^27^ Lee et al. reported MAPbI_3–x_(SCN)_*x*_-based and MAPbI_3_-based gas sensors exhibiting excellent parts per billion-regime (∼200 ppb) H_2_S gas sensing capability.^28^ Ayesh et al. fabricated a high sensitivity H_2_S gas sensor based on FAPbBr_3_ perovskite nanoparticles, which showed a high sensitivity to gas concentrations in the range of 0.5–100 ppm and a fast response time of less than 1 min under ambient room conditions.^29^ However, the toxicity of lead and the instability of lead-based perovskite have limited their applications. Up to date, a H_2_S sensor based on lead-free halide perovskites is still rarely studied.

Cs–Cu–I perovskite, as a type of lead-free perovskite, has shown excellent properties in the field of photoelectric devices due to being environmentally friendly, its high photoluminescence quantum yield, and its excellent air stability.^30,31^ Although 1D CsCu_2_I_3_ and 0D Cs_3_Cu_2_I_5_ have different crystal structures,^32^ they exhibited certain gas-sensitive sensing properties, in addition to ultraviolet light detection properties. For instance, Choi et al. proposed a novel semiconductor and gasochromic multimodal responsive gas sensor based on the gas-induced phase transition mechanism of Cs_3_Cu_2_I_5_.^33^ Sun et al. reported nanostructured-network gas sensors based on lead-free CsCu_2_I_3_, which exhibited excellent room-temperature NO_2_ sensing properties, including an ultralow limit of detection, excellent repeatability, and good selectivity.^34^ However, the detection performance of Cs–Cu–I perovskite materials for other gases has not been reported. Moreover, the underlying mechanisms and electron transport properties are important for understanding those sensor performances. However, the gas detection mechanism of metal halide perovskites is still unclear and needs further study.

In this study, a H_2_S gas sensor utilizing a lead-free CsCu_2_I_3_ film as a sensitive source was developed for the first time. CsCu_2_I_3_ with good air stability is an advantage. The CsCu_2_I_3_ perovskite film was prepared by using a one-step chemical vapor deposition (CVD) process. The gas sensor was fabricated by depositing the CsCu_2_I_3_ film on a glass substrate with prefabricated interdigitated ITO electrodes. The fabricated sensors demonstrated the ability to detect low H_2_S concentrations (∼0.2 ppm) at room temperature under ambient air conditions. Additionally, the possible mechanism for CsCu_2_I_3_ in detecting H_2_S was discussed. This work not only proposed a new preparation method of the H_2_S sensor but also provided a reference for the research of sensing mechanisms of metal halide perovskite.

## 2 Experimental Section

### 2.1 Sensor Fabrication

**Figure 1:**
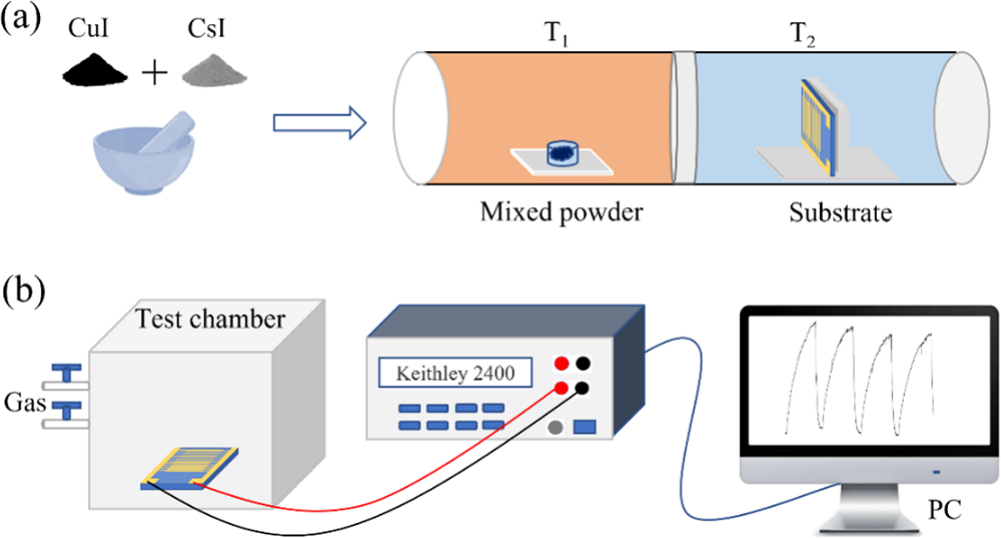
(a) Schematic of the CsCu_2_I_3_ gas-sensor fabrication process. (b) Gas-sensing performance evaluation process.

In the sensor fabrication process, CuI powder (99%) and CsI powder (99.9%) purchased from Sigma-Aldrich were used without any further purification. The preparation diagram is shown in Figure 1a. First, 0.75 mmol CsI and 1.5 mmol CuI were mixed in an agate mortar and grounded for 5 min. The silicon substrate, quartz substrate, and ITO interdigital electrode substrate were ultrasonic-washed with deionized water, acetone, and alcohol successively with 15, 15, and 30 min, respectively. In detail, for the ITO interfinger electrode on the glass substrate, the electrode spacing and electrode width were 50 μm and the interfinger length was 7 mm. Second, the CsCu_2_I_3_ film was prepared by the CVD method with a low vacuum environment (∼1.0 × 10^–3^ Pa), where argon can be used as carrier gas in order to prevent oxidation. The mixed powder was placed upstream in a high-temperature zone at 450 °C, while the cleaned substrates were placed vertically in the low-temperature downstream area set to 150 °C. The holding time of the two temperature zones was 30 min. Finally, the samples were rapidly cooled to room temperature (25 °C).

### 2.2 Material Characterization

The structural properties of the CsCu_2_I_3_ film were analyzed using various techniques. X-ray diffraction (XRD) was performed by scanning the diffraction angle (2θ) from 5.0° to 60.0° with an accuracy of 0.02°. The Cu–Kα1 emission line with a wavelength (λ) of 0.154 nm was used for XRD analysis. The morphology was obtained by scanning electron microscopy (SEM), which provides high-resolution images of the film’s surface. The contents of different elements were carried out by energy dispersive X-ray spectroscopy (EDS). The absorption spectrum of the CsCu_2_I_3_ film was characterized by a UV–vis spectrophotometer. Photoluminescence (PL) and excitation (PLE) spectra were recorded by a fluorescence spectrophotometer (FLS980) with a 450 W xenon lamp source as an excitation source. X-ray photoelectron spectroscopy (XPS) was measured to further explore the compositions and surface properties of the CsCu_2_I_3_ film.

### 2.3 Sensor Performance Evaluation

The gas response of the devices was tested inside a Teflon chamber, where the device was placed in the center without a heating apparatus. The simplified test schematic diagram of gas sensor is shown in Figure 1b. During the test progress, a certain amount of H_2_S gas was injected into the chamber. First, a specific concentration of H_2_S was achieved by injecting a certain amount of H_2_S and air into the gas mixing chamber. Then, the mixed gas was quickly injected into the test chamber. The test chamber was equipped with humidity and temperature sensors to detect the temperature and humidity in real time. The conductivity of the film was changed by the adsorption of H_2_S due to the change in the amount of surface charge. The corresponding electrical signal of the gas response was recorded by a Keithley 2400. All tests are performed at room temperature (∼25 °C) with 45% humidity.

## 3 Results and Discussion

### 3.1 Film Properties' Characterization

**Figure 2:**
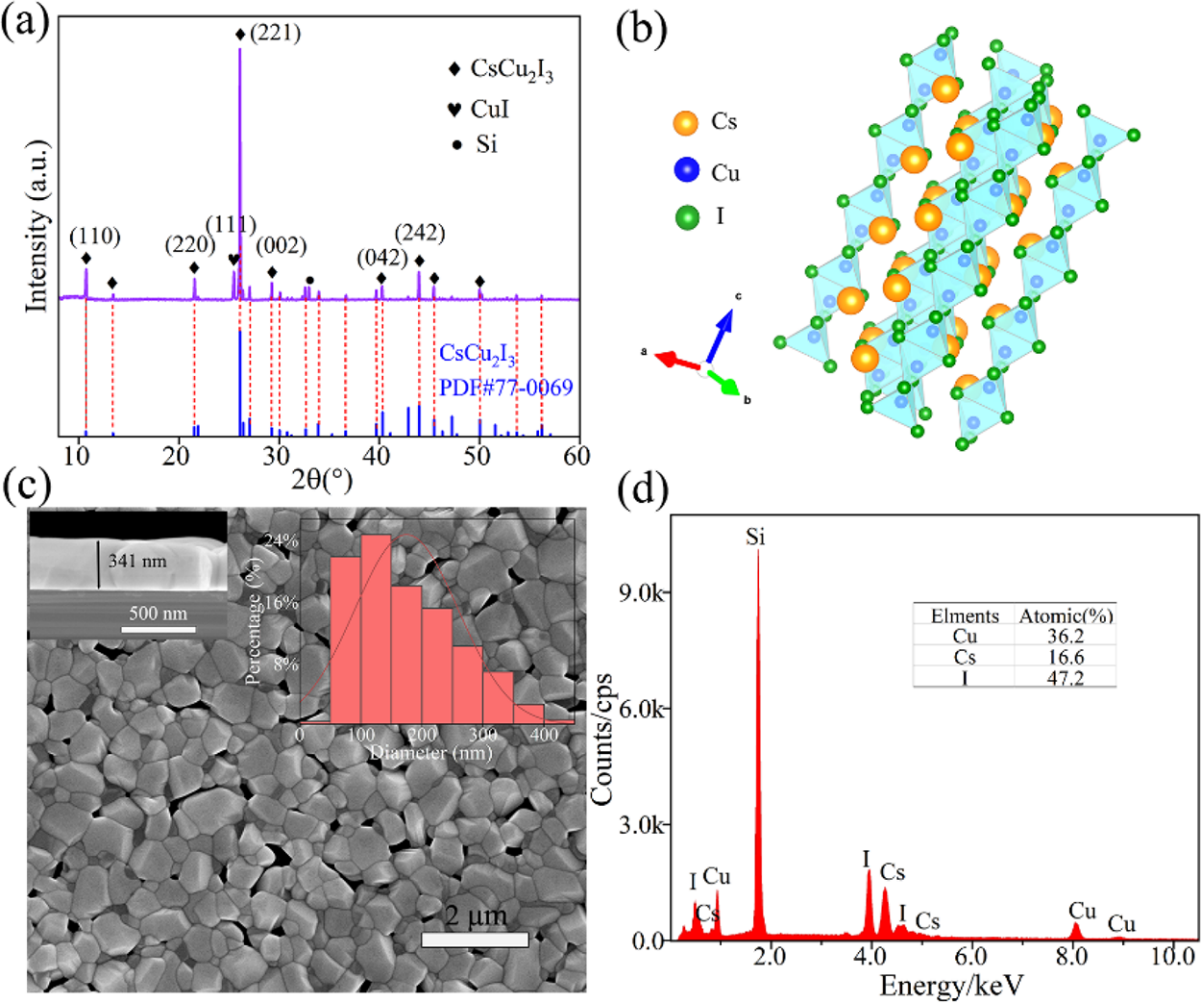
(a) XRD spectrum of the CsCu_2_I_3_ film. (b) Crystal structure diagram of CsCu_2_I_3_ along the [221]. (c) SEM image of as-prepared CsCu_2_I_3_. (d) Elemental analysis from EDS.

As shown in Figure 2a, the XRD pattern exhibited a strong peak at 26.18° and some weak peaks at 10.77°, 21.62°, 29.39°, 40.42°, and 44.10°, which can be attributed to the (221), (110), (220), (002), (042), and (242) crystal planes of CsCu_2_I_3_ (JCPDS: 77-0069), respectively. This outcome of XRD patterns is basically consistent with that reported in the other literatures.^35,36^ Notably, the peak located at 25.45° matches the (111) plane of CuI, indicating that a small amount of CuI exists with CsCu_2_I_3_. The existence of heterophase is a common problem faced by halide perovskites. By the analysis of XRD patterns, the average crystallite size (*D*) can be calculated using the Scherrer formula^37^
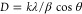
1where *k* = 0.89 and λ = 0.154 nm represent the Scherrer constant and the X-ray wavelength of Cu Kα, respectively. θ is the Bragg diffraction angle, and  represents the structural broadening. β_obs_ and β_std_ are the integral X-ray peak profile width of the sample and a standard of silicon, respectively. It can be calculated that the average crystal size of the prepared CsCu_2_I_3_ film is approximately 87.6 nm based on the (221) plane.

The structure of CsCu_2_I_3_ is well matched with the *Cmcm* space group of an orthorhombic crystal system. The basic unit cell parameters are *a* = 10.54 Å, *b* = 13.17 Å, *c* = 6.09 Å, and α = β = γ = 90°, respectively.^38^ In this representative structure, a Cu ion resides in the center of four I anions, forming the tetrahedral structure of [CuI_4_]^3–^. Two [CuI_4_]^3–^ molecules form a [Cu_2_I_3_]^−^ ionic chain by sharing a common edge, which is combined with Cs^+^ to give one-dimensional CsCu_2_I_3_. The crystal structure of 1D CsCu_2_I_3_ projected along (221) is illustrated in Figure 2b. The SEM image of the CsCu_2_I_3_ film in Figure 2c shows that nanoparticles were uniformly dispersed on the silicon substrate with an average diameter of approximately 174.5 nm by ImageJ software. The size statistics from SEM images are larger than those obtained by XRD calculation. This is due to the convergence of crystalline particles and only the main (221) plane calculated for the XRD values. There were also small interstices between the particles, providing favorable conditions for efficient gas adsorption. The thickness of the film is approximately 341 nm, which can be observed from the cross-section diagram in Figure 2c. EDS analysis confirmed the atomic percentages of Cs, Cu, and I to be 16.6, 36.2, and 47.2%, respectively, which closely match the expected ratio of 1:2:3. The small deviation is attributed to the presence of a small amount of CuI, as observed in the XRD results. Overall, the CsCu_2_I_3_ film obtained by the CVD method exhibited high crystallinity and relatively good purity.

**Figure 3:**
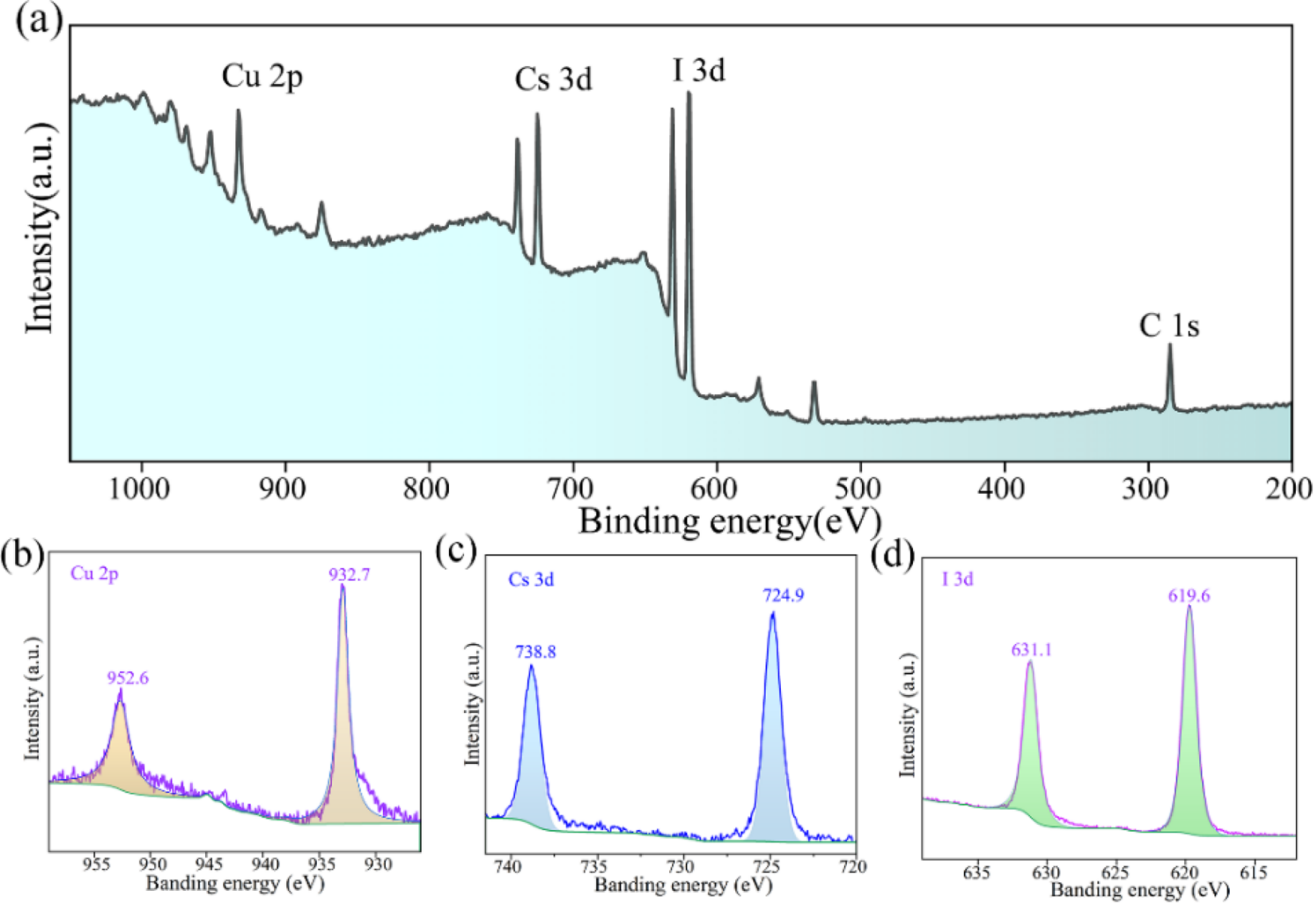
(a) XPS full scan spectrum of the CsCu_2_I_3_ film. High-resolution XPS spectra of (b) Cs 3d, (c) Cu 2p, and (d) I 3d.

As described in Figure 3, XPS measurement is established for the sake of identifying the valency of Cu, Cs, I, and C. The binding energies were corrected using the C 1s peak of the adsorptive carbon, which was measured at 284.5 eV. The presence of Cu 2p, Cs 3d, and I 3d are in a good agreement with the literature.^36,39^ The high-resolution XPS spectra for each constituent element are presented in Figure 3b–d. Two binding energy peaks of Cu 2p are 932.7 and 952.6 eV, respectively, which is consistent with the Cu–I bond and Cu bulk, indicating the presence of Cu^+^. Similarly, two characteristic peaks of Cs 3d at 724.9 and 738.8 eV are attributed to the Cs–I bond and Cs bulk, respectively. The I 3d peaks were observed at 619.6 and 631.1 eV, corresponding to the Cu–I bond and Cs–I bond. The slight deviation in the binding energy is attributed to different environments. These results confirm the stoichiometry of CsCu_2_I_3_, consisting of Cs^+^, Cu^+^, and I^–^, and verify the crystal structure.

**Figure 4:**
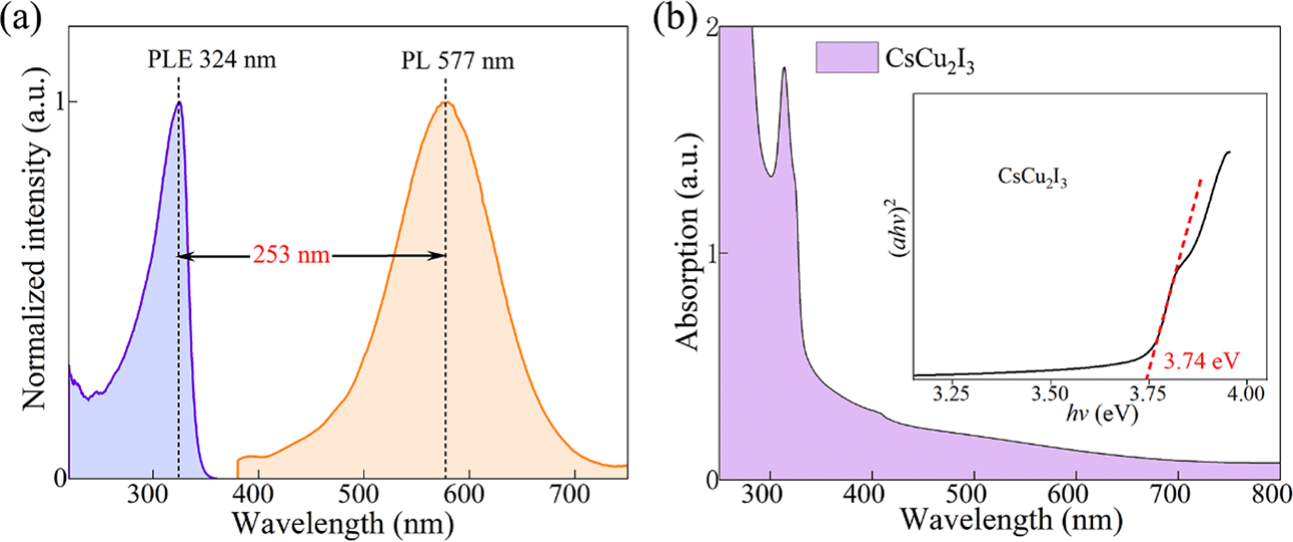
(a) PL and PLE spectra of the CsCu_2_I_3_ film. (b) Absorption of the CsCu_2_I_3_ film, and the inset is the optical band gap.

Additionally, the optical characteristics of the prepared CsCu_2_I_3_ films were analyzed through UV–visible absorption and PL spectra. From Figure 4a, the PLE and PL spectra are located at 324 and 577 nm, respectively, and the half-height width of the broad emission spectrum is ∼112 nm. It is worth noting that there is a large Stokes shift of 253 nm. As can be seen from Figure 4b, the absorption spectrum reveals a distinct edge at 332.4 nm. The band gap is calculated using the following Tauc formula
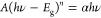
2where *A*, *h*ν, *E*_g_, and α are the proportional constant, photon energy, band-gap energy, and absorption coefficient, respectively. The parameters *n* = 1/2 and 2 represent a direct and indirect band gap, respectively. The CsCu_2_I_3_ film possesses a direct band gap. The calculated value is approximately 3.74 eV. Therefore, the PL mechanism can be elucidated by excited-state structural reorganization or exciton self-trapping rather than a simple direct band-to-band transition.^40,41^ This phenomenon is frequently observed in metal–halide perovskites with low-dimensional structures.^42^ The wide-spectrum yellow-emission characteristics of CsCu_2_I_3_ have a strong potential in the field of light-emitting devices. Similarly, strong absorption in the ultraviolet region can be used for ultraviolet photodetectors.

### 3.2 Gas-Sensing Property

**Figure 5:**
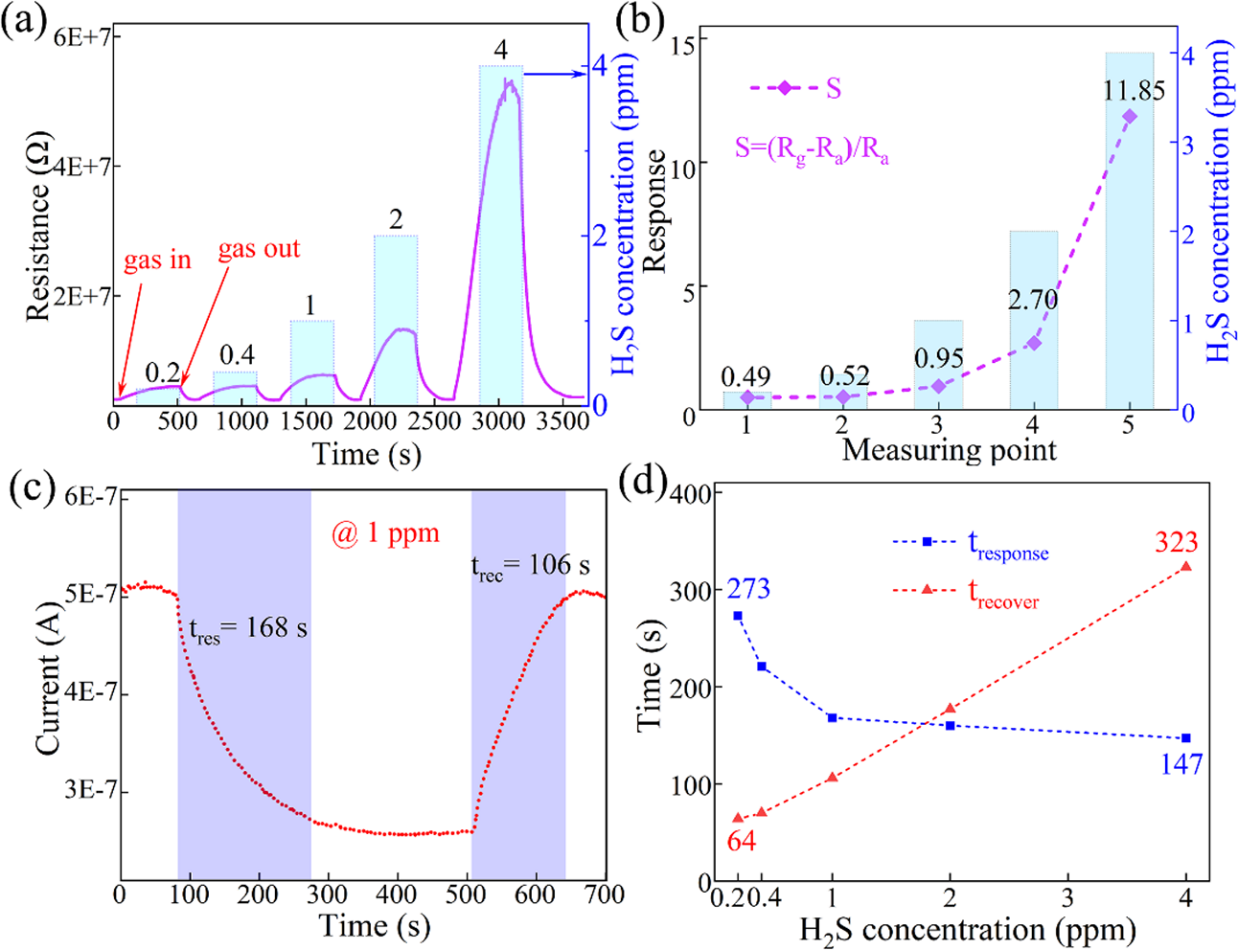
(a) Dynamic resistance curves and (b) response of the CsCu_2_I_3_ gas sensor. Response time and recovery time of the sensor to (c) 1 ppm of H_2_S and (d) different H_2_S concentrations.

In general, the sensing capabilities of semiconductor gas sensors were assessed by keeping track of changes in resistance brought on by gas adsorption. In this study, the dynamic resistance response method was used to assess the CsCu_2_I_3_ film’s H_2_S sensing performance. The sensor was alternately exposed to H_2_S or air at a bias voltage of 2 V. A series of dynamic cycling measurements with gradually increasing H_2_S concentrations onto the sensor were carried out at room temperature with approximately 45% humidity. The corresponding response signals are recorded in Figure 5a. It is found that the resistance of the device increases significantly as the device is exposed to different concentrations of H_2_S. When the H_2_S is exhausted, the resistance of the device returns to the initial state. The higher H_2_S concentration results in a larger resistance. The response value is one of the key parameters to evaluate the H_2_S sensor, which was given by the following formula:
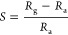
3where *R*_a_ and *R*_g_ are the resistances of the sensor during exposure to air and H_2_S, respectively. As shown in Figure 5b, the increase in H_2_S gas content results in an enhanced response of the CsCu_2_I_3_ sensor. The values of the response were approximately 0.49, 0.52, 0.95, 2.70, and 11.85, corresponding to the H_2_S concentrations of 0.2, 0.4, 1, 2, and 4 ppm. At a low concentration of H_2_S of 0.2 ppm (200 ppb), there is still a strong response close to 0.5, indicating that lead-free CsCu_2_I_3_ materials have great potential in H_2_S gas-sensing sensors. A typical cycling response curve with 1 ppm of H_2_S at room temperature is shown in Figure S1. This exhibits excellent repeatability without response reduction, indicating that the sensor is stable for certain operation periods. The response/recovery time is an important parameter used to evaluate the performance of the gas sensor. The response/recovery time is calculated from the 90% change in resistance then marking the time when the resistance has changed by 90%. The obtained response/recovery time at 1 ppm was approximately 168/106 s, respectively, as described in Figure 5c. Figure 5d depicts the response/recovery time at different H_2_S gas concentrations. The specific response and recovery time curves for different concentrations are shown in Figure S2. It can be seen that the response time is shortened with the increase in the concentration, but the recovery time becomes longer. This is due to the inherent response limitation of the gas sensor. It takes a longer time to reach the limit at lower concentrations. At high concentrations, although more gas is adsorbed in a short time, the time of desorption gas becomes longer. Moreover, the performance of H_2_S gas sensors based on other different materials is compared in recent years, as listed in Table 1.

**Table 1:** Performance Comparison with Recently Reported H_2_S Gas Sensors

material	dynamic range (ppm)	response	OT (°C)	*t*_res_/*t*_rec_ (s)	references
BP/SnO_2_	0.2–9	233.8 at 5 ppm	130	16.4/9.5 at 5 ppm	(43)
BiFeO_3_	0.04–1.2	4.8 at 1.2 ppm	220	3/7 at 10 ppb	(44)
BaSnO_3_	0.1–1.5	16.6 at 1 ppm	150	70/900 at 1 ppm	(45)
FAPbBr_3_	0.5–100	1–2.5	25	60–12/96–84	(29)
MAPbI_3–*x*_(SCN)_*x*_	0.2–10	0.148 at 200 ppb	28.8		(28)
CsPbBr_3_	0.25–100	0.58 at 250 ppb	25	278/730 at 100 ppm	(46)
CsCu_2_I_3_	0.2–4	0.95 at 1 ppm	25	185/106 at 1 ppm	this work

**Figure 6:**
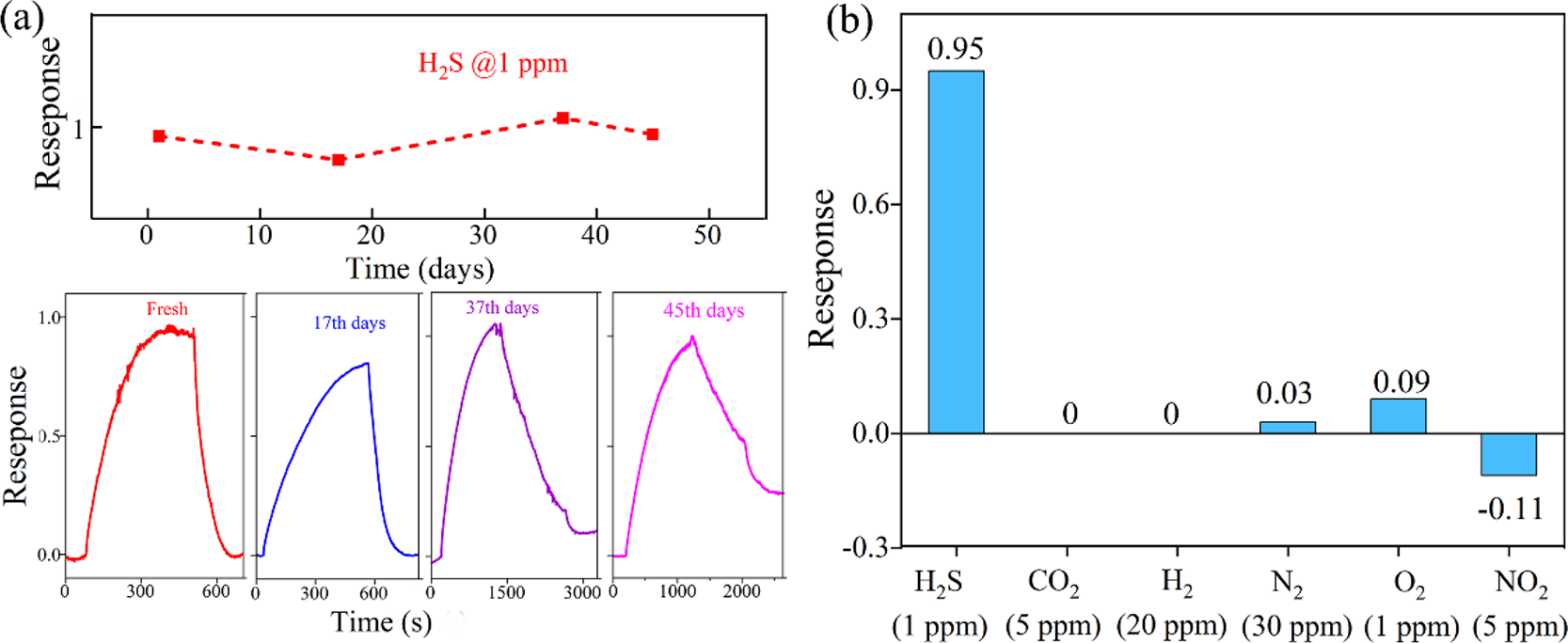
(a) Stability of the sensor-based CsCu_2_I_3_ film; 1 ppm of H_2_S is measured over a 45-day period. (b) Comparison of responses of the CsCu_2_I_3_ film gas sensor to different gases.

In addition, the long-term stability of the gas sensor based on the CsCu_2_I_3_ perovskite film was studied over a period of 45 days at 1 ppm of the H_2_S concentration. The results, as shown in Figure 6a, indicate that the sensor exhibits high stability under ambient moisture and reactive gases. This can be attributed to the inherent stability of the CsCu_2_I_3_ material itself, which is superior to that of lead-based perovskites commonly used in gas sensors. Humidity is another important parameter to the application of gas sensors. A study by Sun et al.^34^ investigated the influence of humidity on a nanostructured CsCu_2_I_3_ sensor for NO_2_ in environments ranging from 11 to 95% relative humidity. The results showed that humidity had a minimal effect on the sensor’s response with a change of less than 0.1% observed.^34^ Due to these findings, detailed studies on humidity were not conducted in the present work. To evaluate the selectivity of the gas sensor, the CsCu_2_I_3_ sensor was exposed to various gases, including CO_2_, N_2_, O_2_, H_2_, and NO_2_. The specific response curves for different gases are shown in Figure S3. As depicted in Figure 6b, the sensor did not exhibit a response to H_2_ and CO_2_, and only a weak response to the two oxidizers was exhibited to O_2_ and N_2_. Notably, the response to NO_2_ was approximately −0.11 at a concentration of 5 ppm, but it remained significantly lower than the response to H_2_S. This negative response value suggests a different response mechanism, which will be discussed in detail in the analysis of the gas-sensitive mechanism. In conclusion, CsCu_2_I_3_ demonstrates good selectivity for H_2_S at low concentrations.

### 3.3 Gas-Sensing Mechanism

The gas-sensing mechanism for metal oxide semiconductor gas sensors has achieved a consensus among researchers.^47,48^ It is a surface-control process that causes obvious changes in the resistance, including three processes of adsorption, redox, and desorption. The core mechanism is the redox reaction of gas. However, in contrast, the gas-sensing mechanism of perovskite materials^25^ is currently in the initial exploration stage, and there is no mature and unified sensing mechanism.

**Figure 7:**
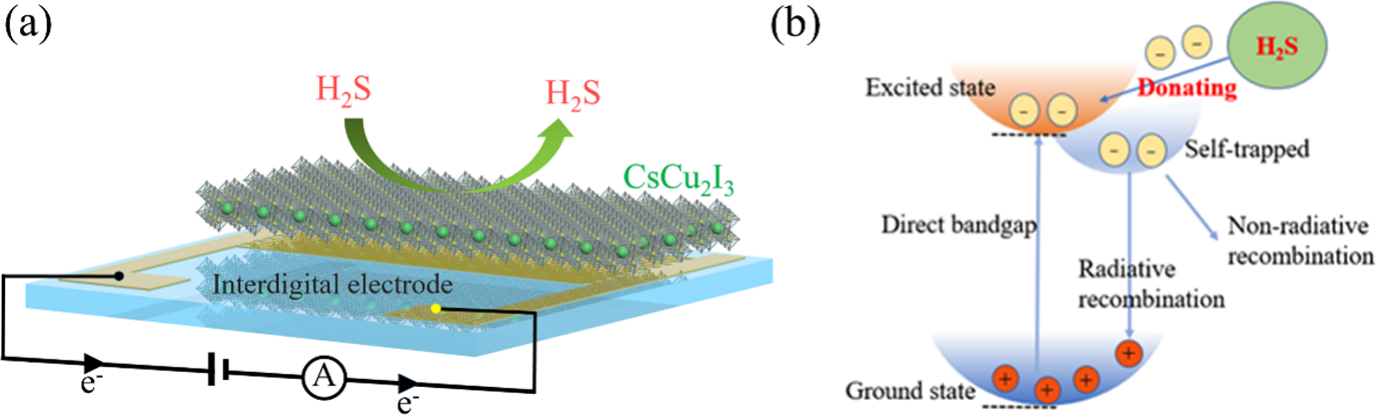
(a) Schematic illustration of the CsCu_2_I_3_ gas sensor; (b) gas-sensing mechanism of CsCu_2_I_3_ for H_2_S gas.

Figure 7a illustrates a schematic of the CsCu_2_I_3_ gas sensor. When the sensor comes into contact with H_2_S gas, the resistance of the CsCu_2_I_3_ film changes, leading to a corresponding change in the current of the device under a specific applied voltage. First, in our opinion, we believe that the defects play a crucial role in the gas sensing properties of perovskite materials. It has been reported that iodine vacancies are commonly present in CuI, CsCu_2_I_3_, and Cs_3_Cu_2_I_5_.^49,50^ According to the EDS results, the atomic percentage of iodine in CsCu_2_I_3_ is less than 50%, indicating the presence of a significant number of iodine vacancies. While defects are usually undesirable in photoelectric devices, they can be beneficial for gas sensors. In the case of halide perovskites, defects can act as electronic traps, enhancing the interaction with gases. Lead-free perovskites, in particular, tend to have more defects compared to lead-based perovskites, which can be advantageous for gas sensing applications.^51^ Additionally, other factors such as dangling bonds and organic ligands in perovskite materials can also create trap states, further increasing the interaction with target gases.^52^ As reported, CsCu_2_I_3_ is a direct band-gap P-type semiconductor that exhibits a large number of self-trapped excitons.^34,53^ When CsCu_2_I_3_ comes into contact with a reducing gas such as H_2_S, the gas acts as an electron donor.^54^ This leads to an increase in minority carriers in the P-type semiconductor, strengthening the nonradiative processes associated with self-trapped excitons. Consequently, the number of available holes decreases, resulting in a decrease in the current, as shown in Figure 7b. Conversely, when CsCu_2_I_3_ contacts an oxidizing gas like NO_2_, the current increases. This conclusion is consistent with the response of the NO_2_ ion in Figure 6b. Therefore, the CsCu_2_I_3_ gas sensor exhibits a tendency of a decreasing current when exposed to H_2_S gas due to the interaction between the gas and the perovskite material, which is facilitated by the presence of defects and trap states as well as the presence of chemical reactions.

**Figure 8:**
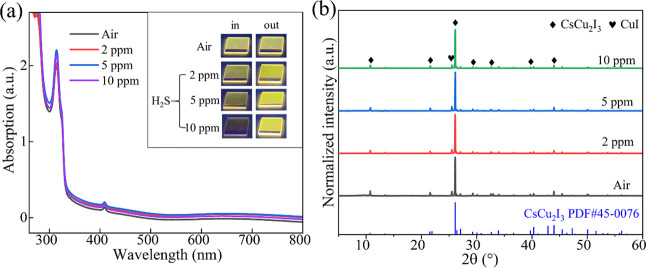
(a) Absorption spectra and (b) XRD of the CsCu_2_I_3_ films in air and after different concentrations of H_2_S were removed. a is a luminescence photograph of samples treated with different concentrations of H_2_S for 5 min.

Additionally, the chemical reaction is another reason. In order to investigate the specific chemical reaction between CsCu_2_I_3_ and H_2_S gas, various characterization techniques were employed. XRD patterns and absorption spectra were used to analyze the CsCu_2_I_3_ film before and after exposure to H_2_S. In this work, the samples were exposed to different concentrations of H_2_S for 5 min and then removed. The sensor was exposed to ambient air as the control group. The color change of the CsCu_2_I_3_ film when exposed to H_2_S gas indicates the occurrence of a chemical reaction. However, when the H_2_S gas is removed, it returns to its previous color. This color change is reversible, as shown in Figure 8a under irradiation of a 254 nm UV lamp. From the absorption spectra in Figure 8a, it does not change significantly when the device recovers. The XRD patterns in Figure 8b also demonstrate that there is almost no difference, indicating that the crystal structure of CsCu_2_I_3_ remains unchanged. Based on these observations, it can be inferred that H_2_S undergoes a reversible chemical reaction with CsCu_2_I_3_, resulting in a reduction in the amount of CsCu_2_I_3_ and a decrease in luminescence. When the H_2_S gas is expelled, the content of CsCu_2_I_3_ returns to its previous state, restoring the previous luminous intensity. As halide perovskites relatively easily adsorb water and H_2_S has high solubility in water, H_2_S gas molecules can react with H_2_O molecules adsorbed on the surface of sensor. The response mechanism can be expressed by the following chemical eqs 4–7:
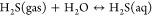
4
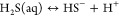
5
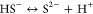
6
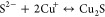
7

Since all of the above reactions are reversible, it is confirmed that the CsCu_2_I_3_ gas sensor can recover when in contact with low concentrations of H_2_S for a short time.

**Figure 9:**
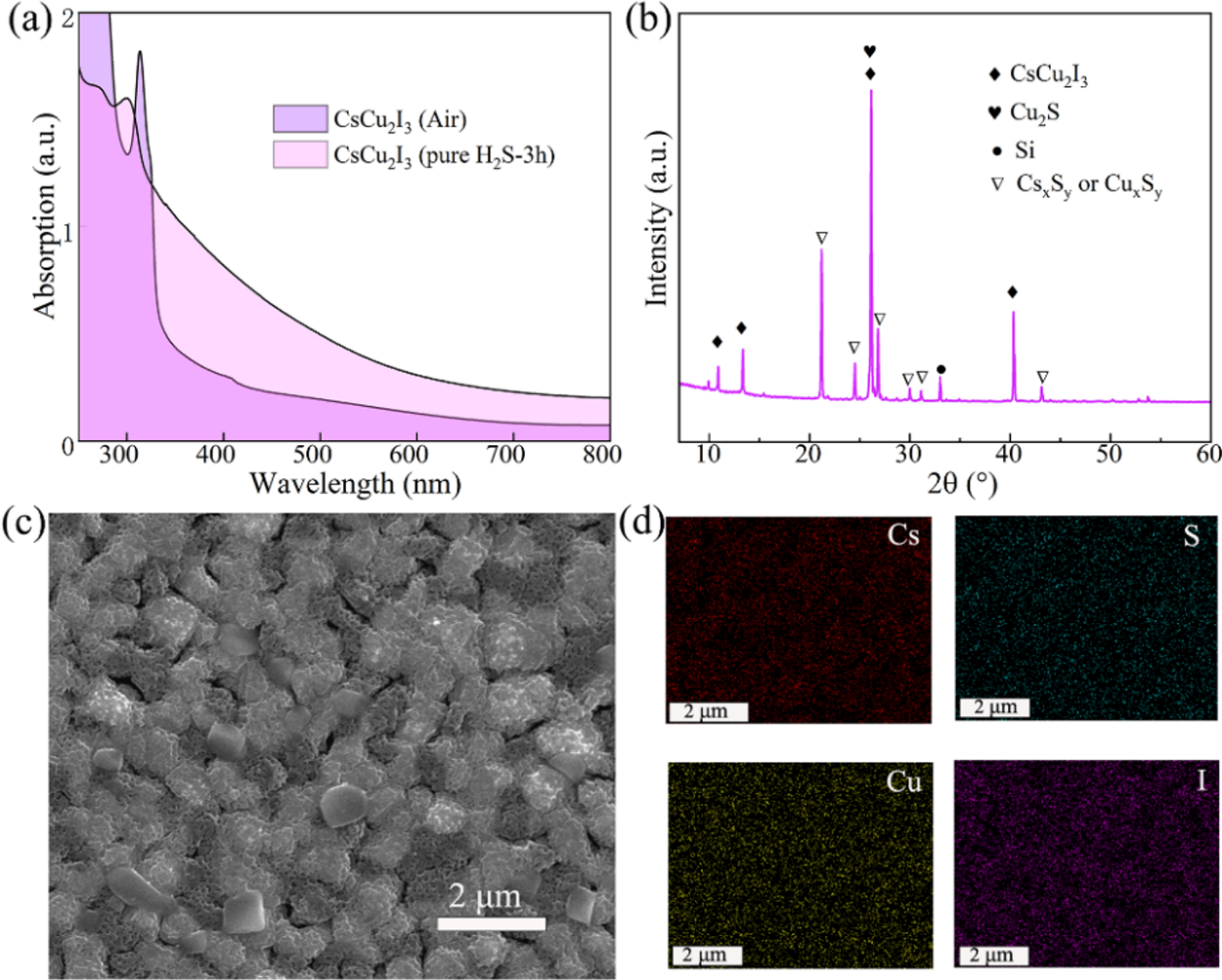
(a) Absorption of the CsCu_2_I_3_ films in air and purity H_2_S for 3 h. (b) XRD patterns, (c) SEM image, and (d) element mapping of the CsCu_2_I_3_ film exposed in pure H_2_S for 3 h.

Additionally, in order to investigate the impact of high concentrations of H_2_S gas for a long period of time on the CsCu_2_I_3_ gas sensor, further testing and analysis were conducted. The sample was exposed in high-purity H_2_S for 3 h and then left for 1 h before being tested. The absorption spectra in Figure 9a show a red shift in the edge of the absorption band. This suggests that the chemical reaction between CsCu_2_I_3_ and H_2_S gas at high concentrations leads to the formation of new compounds. The XRD patterns in Figure 9b also exhibit significant changes after contact with H_2_S gas with the appearance of new crystallization peaks. The peaks at 21.27° and 26.11° correspond to the (111) crystal plane of Cs_2_S and Cu_2_S, respectively. It is indicated that Cu^+^ and Cs^+^ are involved in the reaction. It is worth noting that the (221) plane of CsCu_2_I_3_ and the (001) plane of Cu_2_S are both located at 26.11°, making it difficult to distinguish which crystal plane belongs to which compound. However, the yellow color of the sample in Figure 8a suggests the presence of both Cu_2_S and CsCu_2_I_3_. Additionally, there are also some other crystallization peaks from Cs_*x*_S_*y*_ and Cu_*x*_S_*y*_. This suggests that some irreversible chemical reactions occur with the formation of Cs_*x*_S_*y*_ and Cu_*x*_S_*y*_ compounds. The SEM image of CsCu_2_I_3_ adsorbing pure H_2_S is shown in Figure 9c. This shows that the surface of the crystalline particles becomes rough compared to that in Figure 2c, indicating changes in the morphology of the CsCu_2_I_3_ film. The EDS mapping in Figure 9d confirms the presence of Cu, S, I, and Cs elements that are evenly distributed on the surface. The formation of Cu_2_S and Cs_2_S compounds from some of the Cu^+^ in CsCu_2_I_3_ results in a reduction in the optical band gap of the mixture, leading to the observed red shift in the absorption edge. This further supports the occurrence of a chemical reaction between CsCu_2_I_3_ and H_2_S gas. Therefore, exposure to high concentrations of H_2_S gas can lead to the destruction of the lattice structure of CsCu_2_I_3_ and the occurrence of some irreversible chemical reactions, resulting in changes in the optical properties and morphology of the material.

## 4 Conclusions

In summary, this study successfully presented the development of a H_2_S gas sensor based on a lead-free CsCu_2_I_3_ film prepared through a one-step CVD method. The sensor demonstrated excellent sensing properties, including a low limit of detection, rapid response and recovery times, good repeatability, and high selectivity at room temperature. The sensing mechanism of the gas sensor was discussed in detail, emphasizing the role of defects in perovskite materials as electronic traps, the influence of H_2_S as an electron donor in nonradiative processes associated with self-trapping excitons, and the occurrence of reversible chemical reactions. The findings suggest that lead-free CsCu_2_I_3_ has significant potential for application in H_2_S gas sensors.

## Data Availability

The data that support the findings of this study are available from the corresponding author upon reasonable request.

## Supporting Information Available

Dynamic current of the CsCu_2_I_3_ sensor to 1 ppm of H_2_S; response time and recovery time of the CsCu_2_I_3_ sensor to H_2_S in different concentrations from 0.2 to 4 ppm; and response curves of the CsCu_2_I_3_ film gas sensor to different gases (PDF)
