# Supplementary material for: Highly Sensitive H2S Gas Sensor Based on a Lead-Free CsCu2I3 Perovskite Film at Room Temperature
Source: ACS Omega. 2023 Dec 11;8(50):48326–35. doi: 10.1021/acsomega.3c07694 (PMC10733916; doi:10.1021/acsomega.3c07694)
Supplement: Supplementary file 1 — ao3c07694_si_001.pdf [file ao3c07694_si_001.pdf]

Supporting Information

Highly sensitive H_2S gas sensor based on lead-free $CsCu_2I_3$ perovskite film at room temperature

Kai Ou*, Yue Wang, Wenting Zhang, Yongliang Tang, Yuxiang Ni, Yudong Xia* and Hongyan Wang

School of Physical Science and Technology, Southwest Jiaotong University, Chengdu 610031, Sichuan, China

Corresponding author:

Kai Ou(oukai@swjtu.edu.cn)

Yudong Xia (ydxia@swjtu.edu.cn)

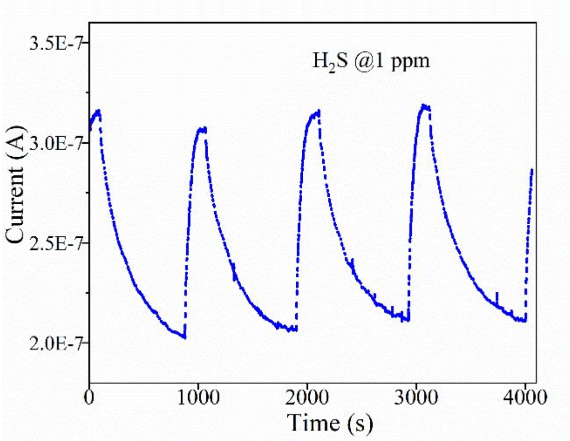

Figure S1. Dynamic current of the $CsCu_2I_3$ sensor to 1 ppm H_2S.

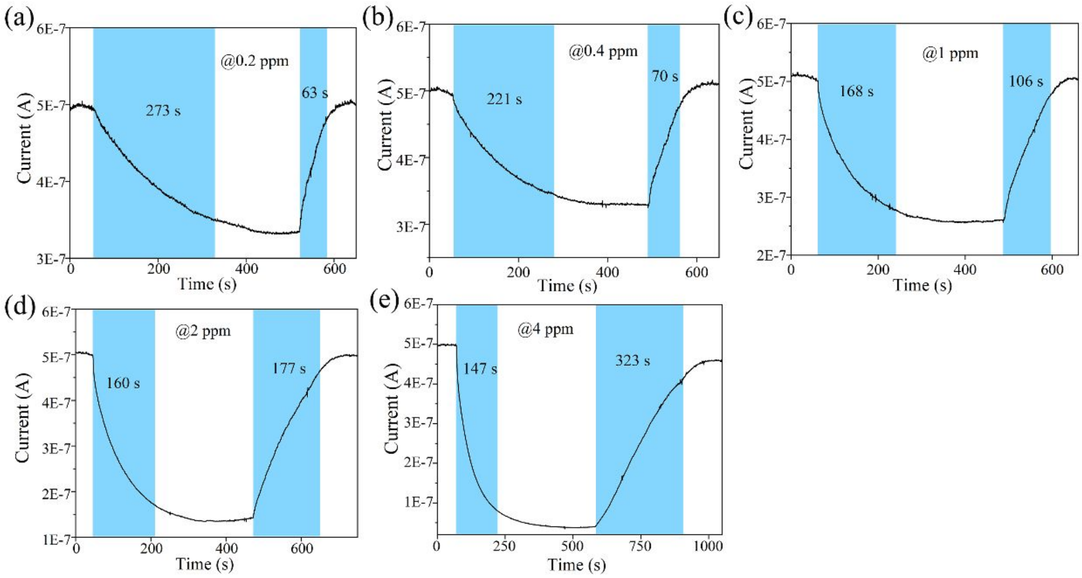

Figure S2. Response time and recovery time of the $CsCu_2I_3$ sensor to H_2S in different concentrations. (a) 0.2 ppm, (b) 0.4ppm, (c) 1 ppm, (d) 2 ppm and (e) 4 ppm.

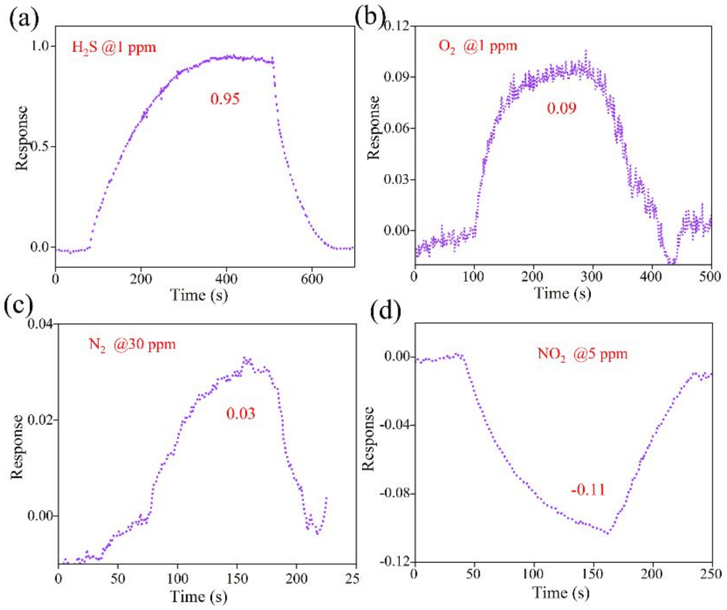

Figure S3. Response curves of the $CsCu_2I_3$ film gas sensor to different gases. (a) H_2S, (b) O_2, (c) N_2 and (d) NO_2.
